# A hybrid approach for chronic pancreatitis: combination of laparoscopic assisted distal pancreatectomy and open Frey procedure

**DOI:** 10.1186/s12893-021-01096-4

**Published:** 2021-02-18

**Authors:** Kenta Saito, Yoichi Matsuo, Goro Ueda, Kan Omi, Yuichi Hayashi, Hiroyuki Imafuji, Ken Tsuboi, Mamoru Morimoto, Ryo Ogawa, Hiroki Takahashi, Itaru Naitoh, Kazuki Hayashi, Hiromi Kataoka, Shuji Takiguchi

**Affiliations:** Department of Gastroenterological Surgery, Nagoya City University Graduate School of Medical Sciences, 1 Kawasumi, Mizuho-cho, Mizuho-ku, Nagoya, Aichi 4678601 Japan

**Keywords:** Chronic pancreatitis, Frey procedure, Laparoscopic distal pancreatectomy, Hybrid approach

## Abstract

**Background:**

The treatment of chronic pancreatitis requires a surgical approach in patients who are refractory to medical therapy. During surgical treatment, ductal decompression is required, but a pancreatectomy is necessary for some patients, such as those with severe stenosis of the pancreatic duct. Indeed, suboptimal procedures lead to recurrent pancreatitis. We used a laparoscopic hybrid approach for patients with severe stenosis of the pancreatic duct. In this report, we present the feasibility and outcomes of our approach.

**Methods:**

We selected a laparoscopic approach for the distal pancreatectomy, which is relatively safe and the effect of reducing the length of the wound is substantial. We selected an open approach for the Frey procedure because complete ductal compression has a high risk for injury to the vessels posterior to the pancreas. We recorded the operative outcomes, postoperative complications, and recurrence of pancreatitis.

**Results:**

We performed the laparoscopic hybrid approach on 3patients between January and December 2018. There were no major intraoperative complications (Clavien-Dindo classification IIIa or more) and the postoperative course was uneventful in all patients. There were no recurrences of pancreatitis and no postoperative pain in all patients in > 2 years of follow-up.

**Conclusion:**

Our hybrid method with a focus on complete ductal compression with safety and minimal invasiveness might be the optimal approach for the surgical treatment of chronic pancreatitis that requires a pancreatectomy with the Frey procedure.

## Background

**Fig. 1:**
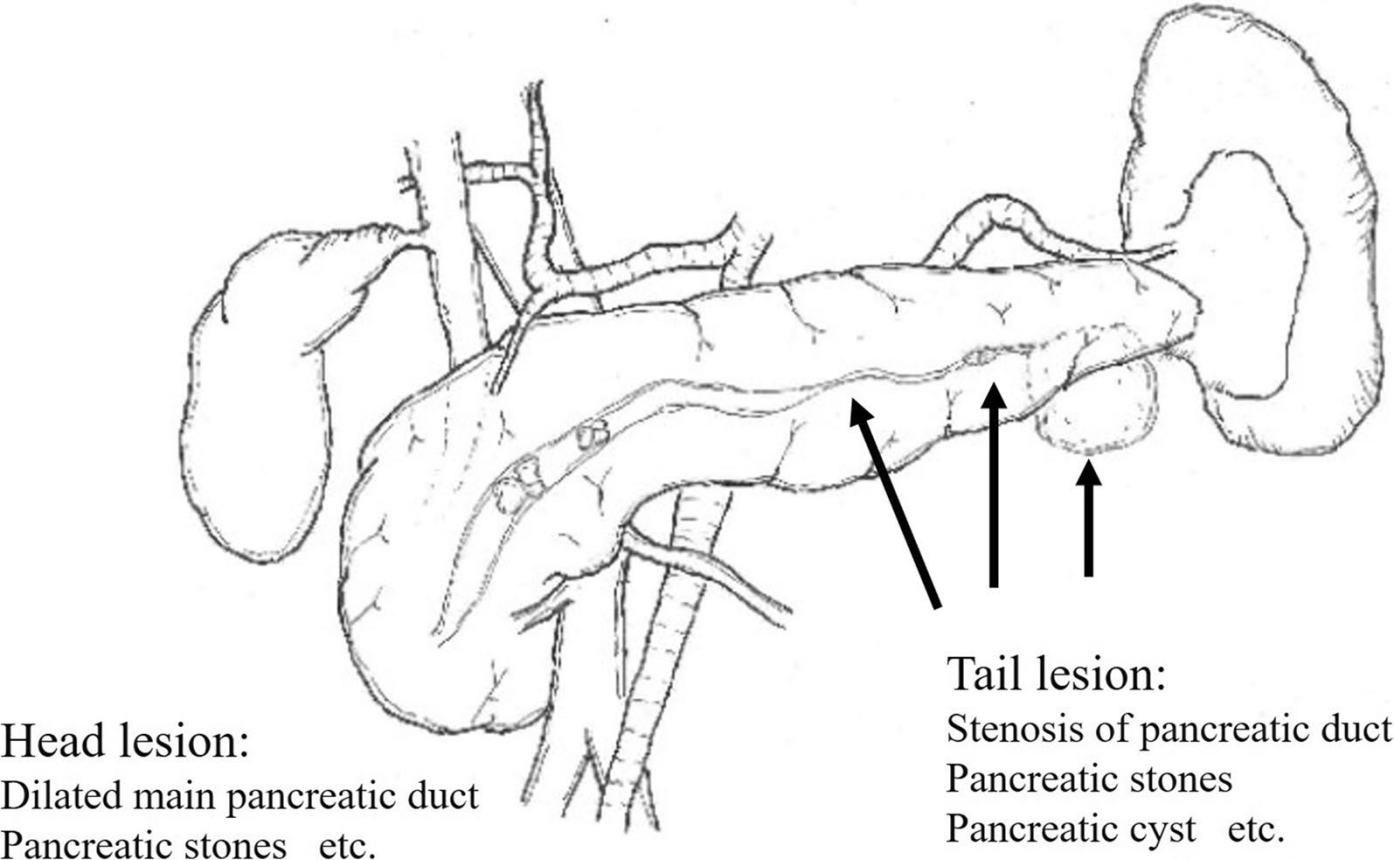
Representative schema of the patient who was suitable for distal pancreatectomy and the Frey procedure for chronic pancreatitis. Dilatation of the main pancreatic duct in the pancreatic head with severe pancreatic tail lesions (arrow), especially stenosis of the main pancreatic duct in the pancreatic body or tail. In this patient, pancreatic duct stenosis frequently caused inflammation and abscess of the distal pancreas

**Fig. 2:**
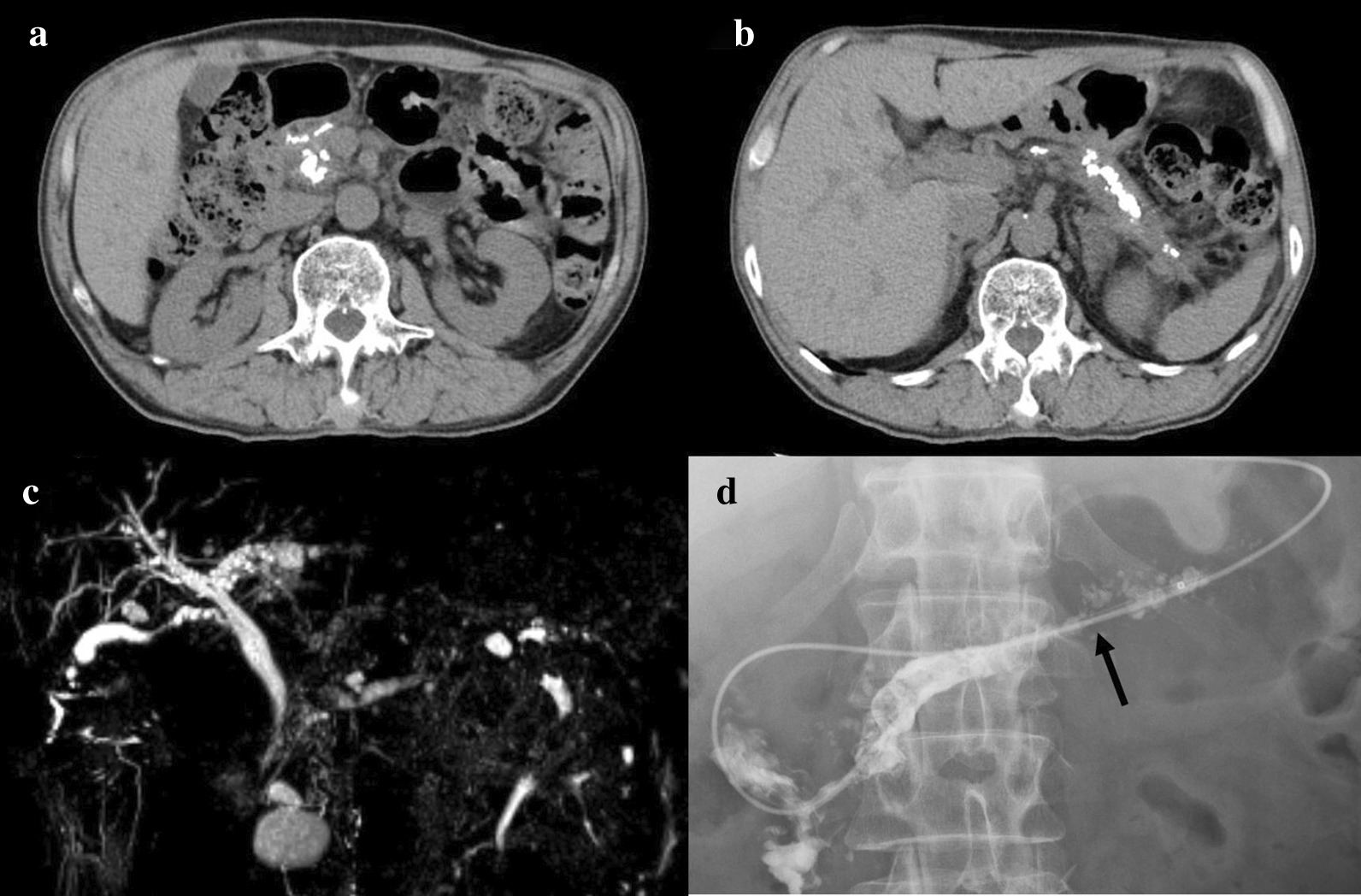
Representative images of the patient who underwent distal pancreatectomy and the Frey procedure for chronic pancreatitis with the hybrid approach. **a**, **b** Computed tomography images are shown. **c** Magnetic resonance cholangiopancreatography is shown. **d** Endoscopic retrograde cholangiopancreatography is shown. Main pancreatic duct was severe stenosis (arrow)

Chronic pancreatitis (CP) includes a wide range of progressive fibro-inflammatory diseases of the pancreas and leads to failure of exocrine and endocrine pancreatic function [1]. Currently, the treatment methods for CP have focused on the management of pain, complications (i.e., duodenal, biliary, and pancreatic obstruction, and pancreatic pseudocysts), and pancreatic insufficiency [2]. The initial treatment for CP is often medical-based therapy, but endoscopic therapy is often performed on patients who are refractory to medical therapy; however, randomized controlled studies have shown that surgery results in significantly greater and more durable pain relief than endoscopic therapy [3, 4]. Many different surgical strategies are used for the various manifestations of CP [5]. The Frey procedure and distal pancreatectomy are efficacious and well-tolerated for CP with dilation of the main pancreatic duct and symptomatic lesions in the pancreatic head and tail (Figs. 1, 2) [6]. Our surgical procedure for CP, which is necessary for distal pancreatic resection, is a hybrid approach involving a laparoscopic-assisted distal pancreatectomy (DP) and an open Frey procedure. From the standpoint of minimal invasiveness, we perform mobilization of the distal pancreas and ligation of the splenic artery laparoscopically. We do not perform the Frey procedure laparoscopically because we are of the opinion that the safest Frey procedure is a hand-assisted approach in which a hand is placed posterior to the head of the pancreas while coring out the head of the pancreas. Herein we report our surgical procedure for patients who require a distal pancreatectomy and the Frey procedure.

## Methods

### Surgical procedures

The patient was placed in the lithotomy position. First, a 12-mm trocar was inserted in the umbilicus using an open method to accommodate a 10-mm rigid laparoscope. Another 12-mm trocar and three 5-mm trocars were then inserted. The abdominal cavity was explored with a pneumoperitoneum maintained at 10 mmHg with CO_2_.

**Fig. 3:**
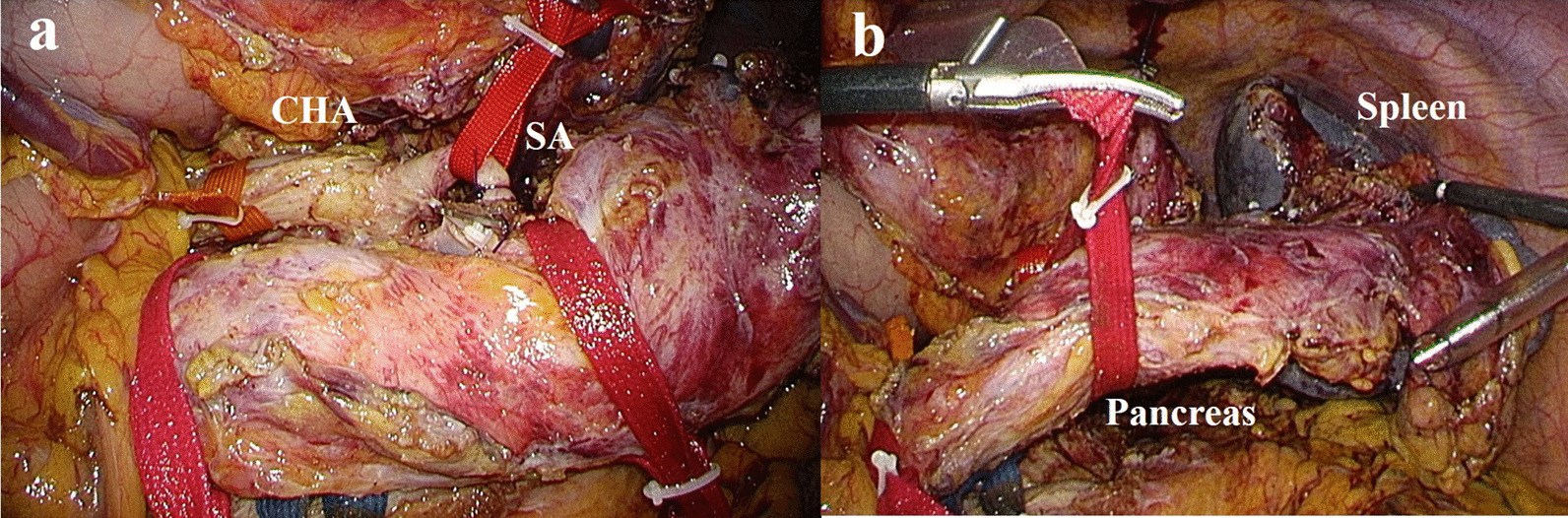
Intra-operative photographs of the laparoscopic approach. **a** Careful dissection and taping of the common hepatic and splenic arteries and ligation of the splenic artery, **b** Mobilization of the distal pancreas and spleen. *CHA* common hepatic artery, *SA* splenic artery

The first step was full mobilization of the spleen and ligation of the splenic vessels, if possible. After dissecting the greater omentum, the gastro-phrenic ligament was transected, including the short gastric vessels, until the upper border of the spleen was confirmed. The stomach was lifted through three tapes after dissecting the lesser omentum. Next, the distal portion of the pancreas was dissected at the lower edge of the pancreas and the splenic flexure of the left colon was mobilized. The dorsal aspect of the pancreas was dissected at the layer of the fusion fascia, but recognition of that layer was often difficult because of inflammation. Finally, we attempted to expose and ligate the splenic vessels. The vessels in patients with chronic pancreatitis are often atherosclerotic, so dissection of the splenic vessels can be risky. In such a case, it is preferable to dissect these vessels after laparotomy to prevent unexpected bleeding in patients with inflammation. In all three cases, we performed ligation of the splenic vessels before the laparotomy. We attribute our ability to do so to the magnifying effect, which facilitated ligation of the splenic vessels. The splenic artery was identified arising from the celiac artery and carefully divided at the roots. The splenic vein was isolated at the bifurcation of the portal vein and encircled by a vessel loop (Fig. 3). Although pancreatic dissection had not yet been performed, most of the steps of the distal pancreatectomy were completed with these procedures.

**Fig. 4:**
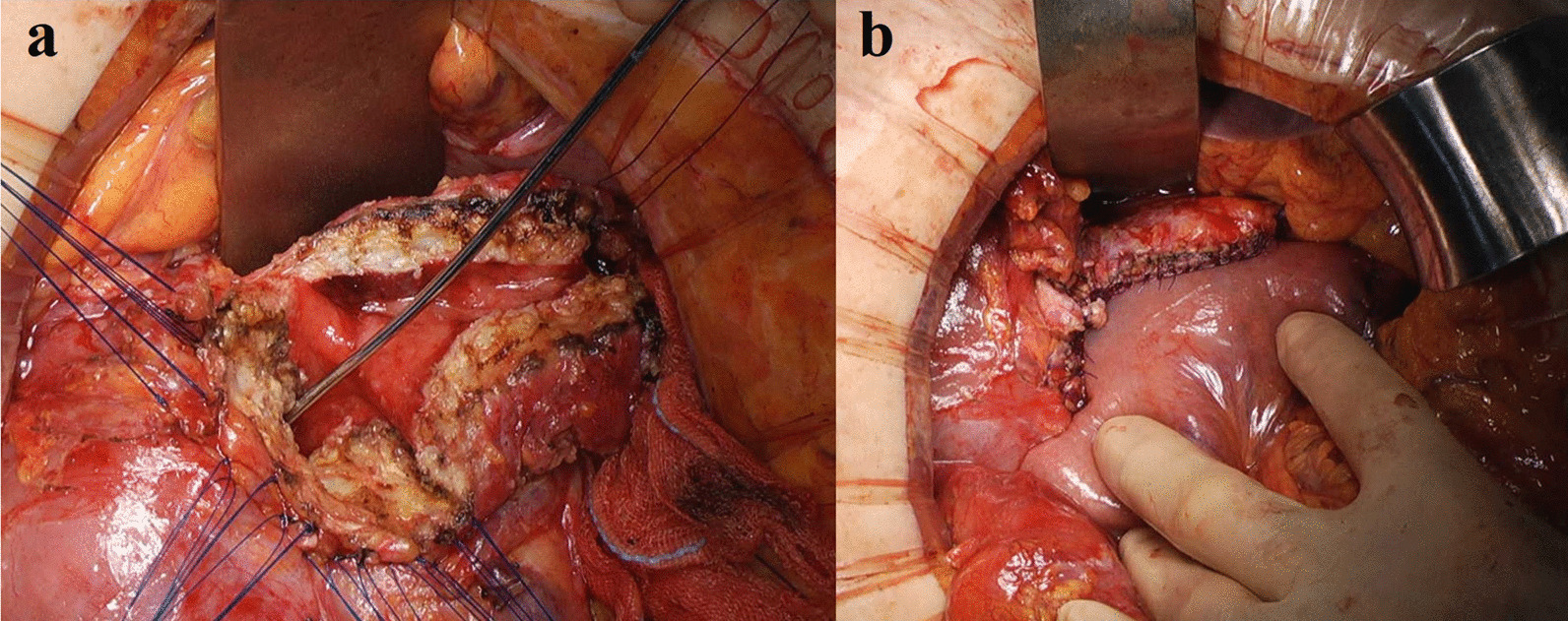
Intra-operative photographs of the open approach. **a** Completion of the frey procedure. **b** Final view after pancreaticojejunostomy

The second step was a laparotomy with a 10-mm incision, then the Frey procedure and pancreaticojejunostomy were performed. A mini-laparotomy incision was made and kocherization was performed to create an adequate space for hand-assisted procedures posterior to the pancreas head. Next, transection of the pancreas and ligation of the splenic vessels were performed. The anterior aspect of the head of the pancreas was exposed in preparation for the Frey procedure and the main pancreatic duct was incised. The gastroduodenal artery was ligated and stitched to prevent bleeding while performing the Frey procedure. Coring out the pancreas and removal of the pancreatic calculus were performed completely with hand assistance posterior to the pancreas to avoid injury to the splenic vein. Finally, a pancreaticojejunostomy was carefully performed while directing attention to cover the stump of the body of the pancreas. Surgical drains were placed at the cranial and caudal ends of the pancreaticojejunostomy (Fig. 4).

## Results

We performed our hybrid procedure on three patients (female, 35 years; male, 59 years; and male, 70 year) between January and December 2018. Alcohol was the etiology of CP in all cases. The average duration of disease from onset was approximately 13 years. The Frey procedure was indicated due to the presence of pancreatic head stones in all cases. Because of the findings in the pancreatic body and tail lesions, we considered it necessary to perform a DP at the same time. Indeed, the indication for a DP in 1 case was the presence of a pancreatic pseudocyst in the pancreatic tail approximately 45 mm in diameter and in 2 cases was severe stenosis of the main pancreatic duct of the pancreatic body. Because the main pancreatic duct could not be fully opened in the two patients with severe stenosis, we performed DPs. The mean operative time was 503 min (laparoscopy, 232 min; open Frey procedure, 271 min), the mean intraoperative blood loss was 474 ml, and there were no blood transfusions required. The mean postoperative hospital stay was 16 days. There was no morbidity or mortality. With > 2 years of follow-up evaluations, there were no recurrences of pancreatitis or postoperative pain. All three patients were relieved of abdominal pain and successfully rehabilitated. With respect to the long-term outcomes, there were no new occurrences of diabetes and the serum albumin levels were normalized after surgery. Specifically, the mean change in serum albumin level before and 1 year after surgery was 0.4 g/dl (preoperative, 4.2 g/dl; postoperative, 4.6 g/dl).

## Discussion

The initial treatment for CP is often medical therapy, but for patients who are refractory to medical therapy, endoscopic or surgical therapy is an alternative. One recent meta-analysis compared endoscopic and surgical interventions in patients with CP and concluded that surgery is a promising approach in the treatment of CP, with the obvious advantage of pain relief, which is difficult to achieve with medical treatment [7]. One study reported that earlier surgical drainage of the obstructed pancreatic duct led to better recovery of histologic changes and pancreatic exocrine dysfunction compared with late surgical drainage in an experimental model of obstructive pancreatitis [8].

A case of CP with severe stenosis of the pancreatic duct required not only the Frey procedure, but also a pancreatectomy (Figs. 1, 2, 3, 4). One study reported that the Frey procedure with a DP can is a promising treatment for CP patients with pancreatic head and tail lesions [6]. We selected a hybrid approach in these cases. Minimal invasiveness is an important consideration in the treatment of CP, which is a benign disease, even though the case is refractory to medical therapy. Benign pancreatic disease is a good indication for laparoscopic surgery [9]. One study reported that laparoscopic distal pancreatectomy is associated with favorable perioperative outcomes compared with open distal pancreatectomy [10]. We selected a laparoscopic approach before performing the Frey procedure, attaching great importance to the minimal invasiveness. The novelty of this procedure is the hybrid approach. An incomplete Frey procedure must be avoided. It is essential that the Frey procedure (coring out of the pancreas and removal of the pancreatic calculus) be completed for treatment of CP. We do not perform these procedures laparoscopically for safety reasons and to ensure completion of the procedure. Indeed, the hybrid procedure is the best approach from the perspective of safety and minimal invasiveness. A procedure based on these concepts is unique.

The strength of our hybrid procedure was the selection of two approaches for the appropriate situation. We selected the laparoscopic approach for situations that could be performed safely with respect to minimal invasiveness and an open approach for situations that are at high risk in terms of safety. For example, the former situation is the DP and the latter situation is the coring out of the head of the pancreas. Our institute performed the hybrid approach for CP, which is necessary for resection of the pancreas with the Frey procedure. We have no experience with an open approach for these cases, thus we compared this new method with the open approach for only Frey and only DP. These results indicated that the perioperative results of our new method were relatively good (Table 1). In our hospital, we basically use a clinical path for pancreatic surgery, and if no complications occur, oral intake will resume on the 5th day after surgery, and the patient will be discharged in approximately 2 weeks in the case of DP. Because the reported cases also used this clinical path, the mean postoperative hospital stay was 16 days.

**Table 1:** Perioperative data of three procedures

	Frey + LapDP (n = 3)	Frey (n = 13)	OpenDP (n = 6)
Operative time, median (range), min	503 (358–637)	496 (284–708)	368 (300–404)
Blood loss, median (range), mL	474 (405–601)	644 (191–3124)	1416 (435–4066)
Pancreatic fistula (GradeB,C), n (%)	0	1 (7.7)	3 (50.0)
Intraabdominal abscess, n (%)	0	1 (7.7)	2 (33.3)
Postoperative bleeding, n (%)	0	0	0
Morbidity (≧Clavien-Dindo Grade IIIa), n (%)	0	2 (15.4)	2 (33.3)
Mortality, n	0	0	0
Postoperative hospital stay, median (range), day	16 (14–19)	23 (11–33)	30 (19–44)

An incomplete Frey procedure is associated with a risk of CP recurrence. The operative time can be long because of the severe inflammation and the need for reliable coring out of the head of the pancreas under such situations. To perform a complete Frey procedure safely, hand assistance is very useful to prevent injury to the splenic vein. We placed our hand posterior to the head of the pancreas to confirm the depth of coring out and the distance to the splenic vein. We are of the opinion that a mini-laparotomy does not add a large incision because a 5-cm wound is needed for extraction of the distal pancreas in any case. We performed the Frey procedure completely to raise the success rate in achieving long-term pain relief.

## Conclusion

Our hybrid approach, with a focus on safety and minimal invasiveness, is best for the surgical treatment of CP, which is necessary for a pancreatectomy and the Frey procedure.

## Data Availability

The datasets used during the current study are available from the corresponding author upon reasonable request.

## Abbreviation

CP: Chronic pancreatitis
